# Microtubule-Mediated NLRP3 Inflammasome Activation Is Independent of Microtubule-Associated Innate Immune Factor GEF-H1 in Murine Macrophages

**DOI:** 10.3390/ijms21041302

**Published:** 2020-02-14

**Authors:** Hsuan-Ju Lai, Yi-Hsuan Hsu, Guan-Ying Lee, Hao-Sen Chiang

**Affiliations:** 1 Department of Life Science, National Taiwan University, Taipei 10617, Taiwan; 2 Genome and Systems Biology Degree Program, National Taiwan University, Taipei 10617, Taiwan

**Keywords:** GEF-H1, microtubule, inflammasome, NLRP3, macrophage, inflammation

## Abstract

Inflammasomes are intracellular multiple protein complexes that mount innate immune responses to tissue damage and invading pathogens. Their excessive activation is crucial in the development and pathogenesis of inflammatory disorders. Microtubules have been reported to provide the platform for mediating the assembly and activation of NLRP3 inflammasome. Recently, we have identified the microtubule-associated immune molecule guanine nucleotide exchange factor-H1 (GEF-H1) that is crucial in coupling microtubule dynamics to the initiation of microtubule-mediated immune responses. However, whether GEF-H1 also controls the activation of other immune receptors that require microtubules is still undefined. Here we employed GEF-H1-deficient mouse bone marrow-derived macrophages (BMDMs) to interrogate the impact of GEF-H1 on the activation of NLRP3 inflammasome. NLRP3 but not NLRC4 or AIM2 inflammasome-mediated IL-1β production was dependent on dynamic microtubule network in wild-type (WT) BMDMs. However, GEF-H1 deficiency did not affect NLRP3-driven IL-1β maturation and secretion in macrophages. Moreover, α-tubulin acetylation and mitochondria aggregations were comparable between WT and GEF-H1-deficient BMDMs in response to NLRP3 inducers. Further, GEF-H1 was not required for NLRP3-mediated immune defense against *Salmonella typhimurium* infection. Collectively, these findings suggest that the microtubule-associated immune modulator GEF-H1 is dispensable for microtubule-mediated NLRP3 activation and host defense in mouse macrophages.

## 1. Introduction

The mammalian immune system uses a variety of pattern recognition receptors (PRRs) located at the extracellular space as well as many subcellular compartments to detect pathogens, host damage signals, or cellular stressors and initiate innate inflammatory responses for host defense [1]. Among these receptors, inflammasomes are cytosolic multi-protein complexes that consist of an inflammasome recognition molecule, the adaptor component ASC (encoded by *Pycard* in mouse), and the effector cysteine protease caspase-1 (encoded by *Casp1* in mouse) [2]. Upon the protein complexes have assembled, the inflammasomes activate caspase-1, which is essential for the proteolytical maturation of pro-IL-1β and secretion of bioactive IL-1β [3]. In addition, inflammasome activation also leads to gasdermin D-mediated pyroptosis, which rapidly limits cell damage and pathogen replication by forming membrane pores [4].

To date, NOD-like receptor family pyrin domain-containing protein 3 (NLRP3) inflammasome is the most intensively described inflammasome since the discovery of inflammasomes in 2002 [2,5]. NLRP3 senses alterations in cellular homeostasis that occur as a result of intracellular microbial infection or accumulation of crystalline materials [6]. Increasing evidence in human data and experimental mouse studies have indicated that the abnormal activation and signaling of NLPR3 inflammasome are responsible for the initiation and progression of deleterious autoinflammatory diseases and common human diseases, such as type 2 diabetes or gout, driven by metabolic dysregulation or formation of crystal aggregations [5,7,8]. Thus, it is critical to identify important interacting or regulatory molecules of NLRP3 activation for the development of potential therapeutics. 

Microtubules belong to the cytoskeleton components that are crucial in innate immunity and cell-autonomous restriction of microbial pathogens in addition to their well-known roles in cell division, migration, and morphology [9]. It has been reported that the disruption of microtubules attenuates NLRP3 inflammasome activation [10,11]. The assembly of NLRP3 inflammasome and adaptor ASC requires the accumulation of acetylated α-tubulin on the microtubules to create optimal sites near the endoplasmic reticulum [10,11]. Further, a centrosomal kinase NIMA-related kinase 7 (NEK7), which controls microtubule nucleation, is an essential component of NLRP3 activation [12,13,14]. Another study also indicates microtubule-affinity regulating kinase 4 (MARK4) governs the positioning of NLRP3 for its activation [15]. Together, these results highlight the importance of microtubule dynamics in the control of NLRP3-driven innate immunity.

The guanine nucleotide exchange factor-H1 (GEF-H1), encoded by *Arhgef2* in mouse, is a microtubule-associated protein that promotes the activation of RhoA guanosine triphosphatases (GTPases) [16]. The activation of GEF-H1 has been linked to several cellular processes, such as cell shape, polarization, differentiation, movement, cell cycle regulation, and epithelial barrier permeability by coupling microtubule dynamics to RhoA GTPase activation [17]. Moreover, alteration of GEF-H1 activity is implicated in the pathogenesis of cancer [18,19]. In addition, GEF-H1 is activated and released from microtubules upon the intracellular binding of bacterial effectors [20,21] and subsequently contributes to the recognition of intracellular bacteria through cytosolic pattern recognition receptors NOD1 and NOD2 [20,22,23]. We have further shown that GEF-H1 mediates the microtubule-dependent sensing of RNA viral infection by RIG-I-like receptors to initiate innate antiviral responses [24]. GEF-H1-deficient mice are more susceptible to influenza virus infection due to severe inflammation in the lungs [24]. These results established the vital role of GEF-H1 in innate immune responses and host defense. However, it is still unclear whether GEF-H1 universally regulates all cytoplasmic PRRs that require microtubules for their activation and subsequent immune responses. 

In this study, we utilized bone marrow-derived macrophages (BMDMs) isolated from wild-type (WT) and GEF-H1-deficient (*Arhgef2^-/-^*, -/-) mice and several inflammasome activators to investigate the molecular contribution of GEF-H1 on the activation of microtubule-dependent NLRP3 inflammasome. We found that GEF-H1 is not crucially involved in NLRP3 inflammasome-mediated IL-1β secretion as well as caspase-1 processing. The acetylation of α-tubulin and mitochondria aggregations do not require GEF-H1 in BMDMs incubated with NLRP3 stimuli. Further, GEF-H1 is dispensable for innate immune defense against *Salmonella typhimurin* infection, which activates the NLRP3 inflammasome. Thus, the microtubule-associated immune modulator GEF-H1 does not contribute to microtubule-mediated NLRP3 inflammasome activation and immune defense in macrophages.

## 2. Results

### 2.1. Dynamic Microtubule Network Controls NLRP3 Inflammasome-Mediated IL-1β Production

**Figure 1:**
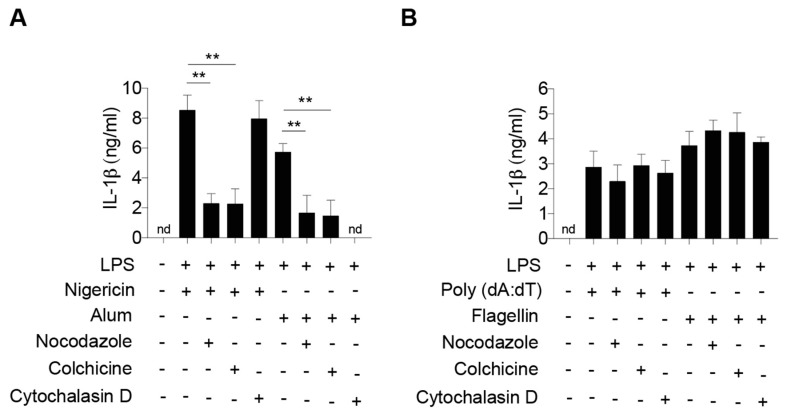
Disruption of the microtubule network suppresses the activation of NLRP3 inflammasome. ELISA of IL-1β in culture supernatants of LPS-primed bone marrow-derived macrophages (BMDMs) pretreated with or without microtubule or actin disruptors followed by (**A**) NLRP3, (**B**) AIM2 or NLRP4 inflammasome inducers. *n* = 3 independent experiments per group. Data represent the mean ± SD. Statistical analyses were performed using one-way ANOVA with Turkey’s multiple comparisons. **, *p* < 0.01.

To confirm that the microtubule network mediates NLRP3 inflammasome activation, we stimulated BMDMs isolated from WT C57BL/6 mice with chemicals (nocodazole or colchicine) that inhibit microtubule polymerization followed by determining the expression of IL-1β in response to combinations of lipopolysaccharide (LPS) and various NLRP3 stimuli. Consistent with previous studies [10,11], activation of NLRP3 inflammasome by soluble stimulus nigericin or particulate matter alum resulted in robust secretion of Il-1β from WT BMDMs, which was attenuated in cells incubated with microtubule-disrupting nocodazole or colchicine (Figure 1A). In contrast, incubation of nocodazole or colchicine in WT macrophages did not suppress the production of Il-1β in response to flagellin, an NLR family CARD domain-containing protein 4 (NLRC4) inflammasome activator or an absent in melanoma-2 (AIM2) inflammasome activator double-stranded DNA poly(dA:dT) (Figure 1B). Moreover, disruption of the actin network, another major cellular cytoskeleton, by cytochalasin D treatment only suppressed the alum activation of NLRP3 inflammasome that requires functional phagocytosis pathways [25]. In contrast, disruption of actin network prevented neither phagocytosis-independent stimulus-mediated IL-1β secretion of NLRP3 inflammasome nor NLRC4 inflammasome or AIM2 inflammasome in WT macrophages (Figure 1). In summary, these results highlight the specific role of the microtubule network in the activation of NLRP3 inflammasome in response to both soluble and insoluble inducers.

### 2.2. Deficiency of GEF-H1 Does Not Affect the Secretion of IL-1β in Response to NLRP3 Inflammasome Inducers

**Figure 2:**
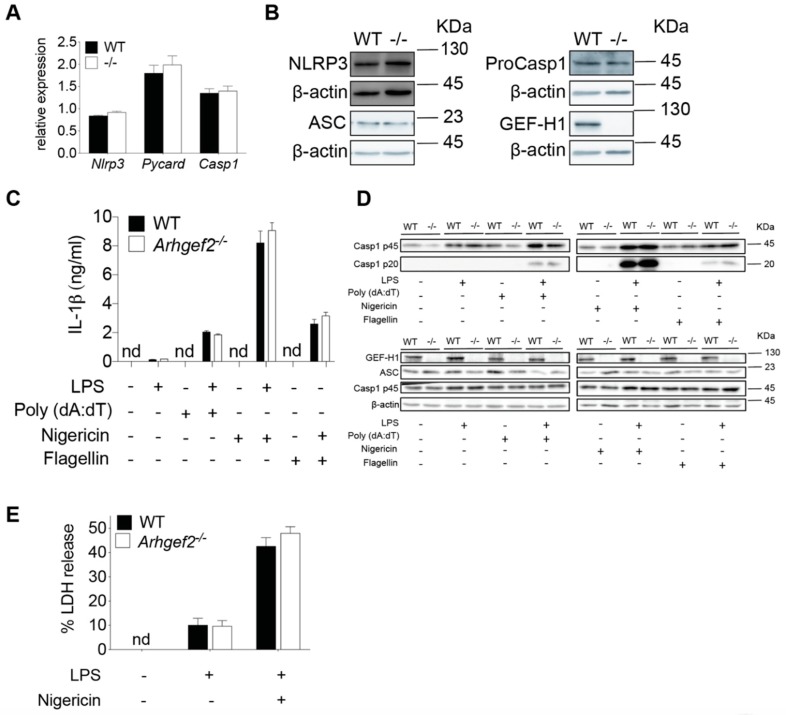
GEF-H1 is not required for NLRP3-mediated IL-1β secretion, capase-1 processing, and LDH release in LPS-primed macrophages incubated with nigericin. (**A**) Quantitative RT-PCR analysis of *Nlrp3*, *Pycard*, and Casp1 mRNA expression in untreated wild-type (WT) and *Arhgef2^-/-^* bone marrow macrophages. Values are normalized to the expression of *Gapdh*. (**B**) Representative western blot analysis of NLRP3, ASC, and Pro-Caspase-1 (ProCasp1) in untreated WT and *Arhgef2^-/-^* (-/-) BMDMs. (**C**) IL-1β released by LPS-primed WT and *Arhgef2^-/-^* BMDMs treated with NLRP3, AIM, or NLRC4 inflammasome inducers. (**D**) Representative western blot analysis of pro-caspase-1 (Casp1 p45), active-caspase-1 subunit p20 (Casp1 p20), and ASC in the supernatants or cell lysates of LPS-primed WT and *Arhgef2^-/-^* (-/-) BMDMs left unstimulated or incubated with indicated inflammasome activators. (**E**) LDH released by LPS-primed WT and GEF-H1-deficient BMDMs incubated with nigericin. *n* = 3 independent experiments per group. Data represent the mean ± SD. Statistical analyses were performed using an unpaired two-tailed *t*-test.

Colchicine is an effective and frequently used drug that inhibits microtubule polymerization for the treatment of gout flare [26]. Notably, colchicine has been shown to attenuate the production of IL-1β in response to MSU-induced NLRP3 activation [10]. Given microtubule-associated immune molecule GEF-H1 is released upon the treatment of microtubule destabilization agent colchicine [17,19], this prompted us to perform a detailed investigation of NLRP3 inflammasome activation in WT and GEF-H1-deficient macrophages. We first examined the expression of core NLRP3 inflammasome components in both WT and GEF-H1-deficient BMDMs. The expression level of NLPR3, ASC, and proCaspase-1 mRNA (Figure 2A) and protein (Figure 2B) was similar in macrophages derived from *Arhgef2^-/-^* mice and wild-type littermates before stimulation, demonstrating GEF-H1 was not involved in the expression of NLRP3 inflammasome components at the transcriptional and translational level. We then examined NLRP3 inflammasome activation in WT and *Arhgef2^-/-^* bone marrow macrophages primed with LPS followed by nigericin stimulation. As a specificity control, we also determined the activation of NLRC4 inflammasome and AIM2 inflammasome in WT and GEF-H1 knockout macrophages that are known to be independently of microtubule network [11]. As expected, the level of IL-1β production was comparable between WT and GEF-H1 knockout BMDMs after incubation with stimuli, such as flagellin and poly(dA:dT) known to induce microtubule-independent inflammasome pathways (Figure 2C). Treatment of LPS-primed macrophages with NLRP3 inflammasome activator led to IL-1β secretion (Figure 2C). Surprisingly, upon NLRP3 activation, we did not observe any significant differences in IL-1β secretion among WT and GEF-H1-deficient macrophages (Figure 2C). Inflammasome-mediated IL-1β is synthesized as an inactive precursor (pro-IL-1β) and requires post-translational processing by caspase-1 for the generation of mature and active IL-1β [3]. Upon inflammasome activation, inactive pro-caspase-1 is converted to active caspase-1 via dimerization, followed by autocatalysis that generates an active molecule composed of two large and two small subunits [3]. We observed that the activation of caspase-1 was also nearly identical between WT and *Arhgef2^-/-^* BMDMs as determined by caspase-1 cleavage and secretion (Figure 2D). Further, NLRP3-mediated caspase-1 activation leads to pyroptosis in macrophages that can be measured by the released lactate dehydrogenase (LDH) activity in the cell culture supernatants. Consistent with the pattern of caspase-1 processing, the level of LDH release of primed WT and GEF-H1-deficient macrophages in response to nigericin was comparable (Figure 2E). Together, these results suggest that NLRP3-mediated IL-1β secretion, caspase-1 processing, and cell death were not regulated by GEF-H1 expression in mouse macrophages.

### 2.3. GEF-H1 Is Not Required for α-Tubulin Acetylation and Mitochondria Redistribution in LPS-Primed Macrophages Incubated with NLRP3 Inducer

**Figure 3:**
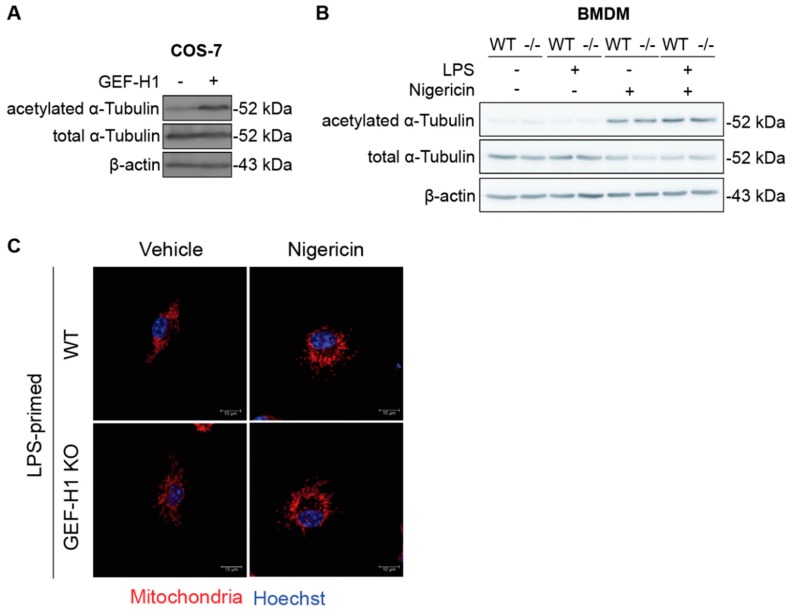
GEF-H1 is not involved in α-tubulin acetylation and mitochondria redistribution in LPS-primed macrophages incubated with nigericin. (**A**) Representative western blot analysis of acetylated α-tubulin in GEF-H1-overexpressed COS-7 cells. (**B**) Representative western blot analysis of α-tubulin acetylation in LPS-primed BMDMs left unstimulated or stimulated with nigericin. (**C**) Representative immunofluorescence images of mitochondria distribution in LPS-primed BMDMs left unstimulated or stimulated with nigericin. Data are from one experiment representative of three independent experiments.

Microtubules are the well-known platforms for mediating the intracellular transport of various organelles [27]. Published studies have shown that microtubule α-tubulin acetylation is an essential regulator of NLRP3 activation [11]. Moreover, microtubule acetylation promotes the accessibility of microtubule-associated proteins to microtubules [28]. We first transfected plasmids encoding GEF-H1 in COS-7 cells and examined the level of α-tubulin acetylation (Figure 3A). GEF-H1 overexpression led to increased acetylation of α-tubulin, indicating that GEF-H1 may involve in the regulation of α-tubulin acetylation in COS-7 cells. To extend the previous observation that GEF-H1 does not influence NLRP3 inflammasome activation, we determined if GEF-H1 is required for the acetylation of α-tubulin during NLRP3 activation in macrophages. Interestingly, western blot analysis did not show any alteration of total or acetylated α-tubulin in LPS-primed GEF-H1-deficient BMDM in response to NLRP3 inflammasome activator when compared to WT BMDM (Figure 3B), suggesting that GEF-H1 was not essential or required for microtubule acetylation in mouse macrophages.

It has been reported that acetylation of α-tubulin controls the assembly of the inflammasome complex, particularly NLRP3 [11]. In addition, microtubule motor protein dynein facilitates ASC on mitochondria moving toward NLRP3 on the endoplasmic reticulum in response to NLRP3 inflammasome activators [11]. Considering GEF-H1 binds to the dynein motor on microtubules [29], we therefore addressed whether GEF-H1 affects mitochondrial distribution in macrophages with or without NLRP3 inflammasome inducers. Immunofluorescence staining showed that mitochondria evenly distributed in the cytosol of both unstimulated WT and GEF-H1-deficient macrophages. Treatment of nigericin further promoted the aggregation of mitochondria located at the perinuclear region of WT and GEF-H1-deficient macrophages, indicating GEF-H1-deficiency did not affect the localization and distribution of mitochondria upon NLRP3 inflammasome activation (Figure 3C). Together, our results suggested that GEF-H1 is not involved in the acetylation of α-tubulin nor the redistribution of mitochondria in LPS-primed macrophages incubated with NLRP3 inducer nigericin.

### 2.4. GEF-H1 Is Dispensable for the Immune Defense against Salmonella typhimurim Infection in Macrophages

**Figure 4:**
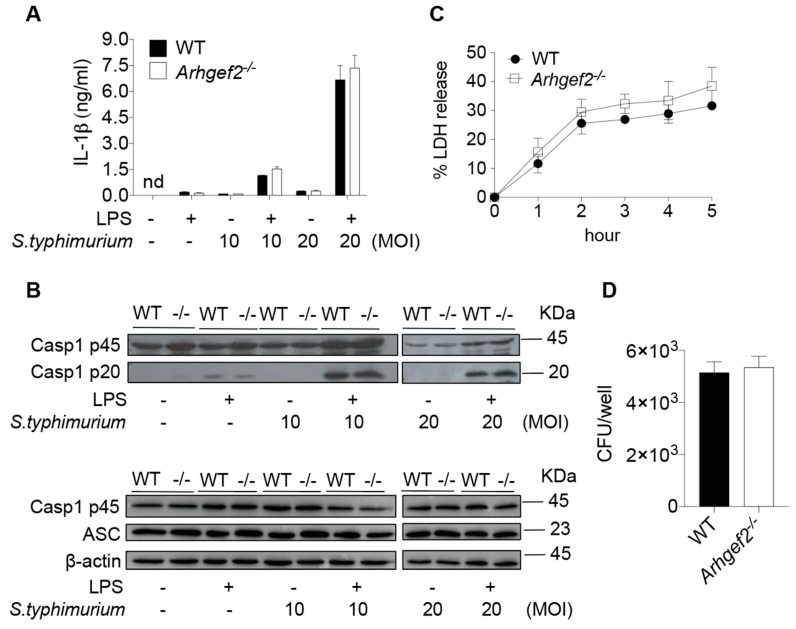
GEF-H1 has minimal impact on immune defense against *S. typhimurium* infection. (**A**) IL-1β released by LPS-primed WT and GEF-H1-deficient BMDMs infected with *S. typhimurim* at different MOI. (**B**) Representative western blot analysis of pro-caspase-1 (Casp1 p45), active-caspase-1 subunit p20 (Casp1 p20), and ASC in the supernatants or cell lysates of LPS-primed BMDMs left unstimulated or infected with *S. typhimurim* at indicated MOI. (**C**) LDH released by LPS-primed WT and GEF-H1-deficient BMDMs infected with *S. typhimurim* (MOI = 10) at different time points. (**D**) Intracellular CFU was measured by a gentamicin protection assay in LPS-primed WT and GEF-H1-deficient BMDMs infected with *S. typhimurim* at MOI = 10 for 3 h. *n* = 3 independent experiments per group. Data represent mean ± SD. Statistical analyses were performed using unpaired two-tailed *t*-test.

Thus far, we found no evidence of the involvement of microtubule-associated GEF-H1 in the activation of NLRP3 inflammasome. In addition to the maturation and secretion of IL-1β, the activation of inflammasomes also mounts an innate immune defense against microbial infection in macrophages. To further assess the role of GEF-H1 in inflammasome-mediated host defense, we subjected WT and *Arhgef2^-/-^* BMDMs to *Salmonella* pathogenicity island 1 (SPI-1) type 3 secretion system (T3SS)-independent *Salmonella enterica* serovar Typhimurium *(S. typhimurium)* infection that drives activation of the NLRP3 inflammasome [30]. *S. typhimurim* grown to stationary phase, which resulted in the reduced expression of SPI-1 [31], elicited similar amounts of IL-1β released from cultured WT and *Arhgef2^-/-^* BMDMs at 16-h post-infection (Figure 4A). The extent of IL-1β secretion was correlated with the processing of capsase-1 to its active form caspase-1 p20 subunit as determined by western blotting in the culture supernatants (Figure 4B) of WT and GEF-H1-deficient macrophages. Moreover, WT and *Arhgef2^-/-^* BMDMs contained similar numbers of bacteria at 3-h post-infection (Figure 4C). Similarly, we did not observe any difference in LDH release between WT and GEF-H1-deficient macrophages upon *S. typhimurim* infection (Figure 4D). Together, these results indicate that GEF-H1 is not critical for NLRP3-mediated immune defense against *S. typhimurim* infection in macrophages.

## 3. Discussion

In the current study, we aimed to evaluate the impact of GEF-H1 and cell cytoskeletons on NLRP3 inflammasome activation in mouse macrophages. We demonstrate that the activation of NLRP3 inflammasome depends on intact microtubule networks. In contrast, neither AIM2 inflammasome nor NLRC4 inflammasome requires microtubules for IL-1β secretion. We also showed that the actin network is required for phagocytosis-dependent activation of NLRP3 inflammasome. In contrast, the actin network is dispensable for phagocytosis-independent NLRP3, NLRC4, and AIM2 inflammasome activation in BMDMs. Furthermore, GEF-H1 deficiency has minimal impact on NLRP3-driven IL-1β maturation and caspase-1 processing as well as NLRP3-mediated immune response against *S. typhimurium* infection in mouse bone marrow macrophages.

The importance of microtubule dynamics has been suggested in the initiation of RLR-mediated [24] or NLRP3-dependent innate immune responses [10,11] in mouse macrophages. While the microtubule-associated immune modulator GEF-H1 is crucial for RLR-mediated signaling [24], data presented in the current study suggest that GEF-H1 is not critically involved in the regulation of NLRP3 activation and immune defense. Moreover, several studies have indicated that α-tubulin acetylation facilities the NLRP3 inflammasome assembly and activation [11,32]. Although we did find the overexpression of GEF-H1 in COS-7 cells leads to increased α-tubulin acetylation, this post-translational modification of microtubules is independent of GEF-H1 for NLRP3 inflammasome activation in macrophages incubated with NLRP3 inducers. Furthermore, we also demonstrated that the cell cytotoxicity of WT and GEF-H1-deficient macrophages in response to NLRP3 inducers and *S. typhimurium* are comparable. GEF-H1 may therefore minimally impact the activation of NLRP3-dependent gasdermin D for pryoptosis in macrophages. Thus, one can anticipate that the activation of NLRP3 inflammasome more likely depends on other microtubule-associated proteins, such as microtubule-affinity regulating kinase 4 (MARK4) [15].

Whether GEF-H1 controls the activation of other inflammasomes remains to be explored. Similar to what we observed that colchicine abrogates NLRP3-dependent IL-1β release, a recent study has found that colchicine also suppresses the release of pyrin inflammasome-mediated IL-1β in a RhoA-dependent manner [33,34,35]. Given that colchicine treatments are known to induce the release of GEF-H1 from microtubules [19] and GEF-H1 is the specific activator for RhoA [16,17], it would be critical to examine whether GEF-H1 regulates the pyrin inflammasome activation that plays a critical role in pyrin-mediated autoinflammatory disorders such as familial Mediterranean fever (FMF) and hyperimmunoglobulinemia D syndrome (HIDS) [33,36]. Intriguingly, upon the pyrin inflammasome activation, GEF-H1-deficient BMDMs secreted significantly higher IL-1β in the cell supernatants when compared to those of WT BMDMs suggesting that GEF-H1 may play a regulatory role in the pryin inflammasome activation (Supplementary Figure S1). A more detailed analysis of the interaction among the pyrin inflammasome, RhoA, and GEF-H1 is required to further delineate the underlying mechanisms. 

Inflammasome-mediated innate immune response is critical for host defense against *S. typhimurium* because mice lacking Nlrp3, Nlrc4, caspase-1, and IL-1β are highly susceptible to *S. typhimurium* infection [30,37,38]. *S. typhimurium* translocates bacterial virulence factors and small amount of flagellin by type III secretion system (T3SS) to induces capase-1 activation via both the NLRP3 inflammasome [30] and NLRC4 inflammasome [39]. In the current study, we have observed that the deficiency of GEF-H1 does not affect the immune response and susceptibility of BMDMs in response to S. *typhimurium* infection. These results further establish the dispensable role of GEF-H1 in innate immune defense against S. *typhimurium* infection in murine macrophages. 

Together, our studies unravel a dispensable role of GEF-H1 in regulating NLRP3 inflammasome activation and immune defense against *S. typhimurium* in mouse macrophages. Future work is required to identify the critical molecules that differentially control either microtubule-based or microtubule-independent inflammasome activation for developing better therapeutic strategies to inflammasome-mediated inflammatory diseases. 

## 4. Materials and Methods

### 4.1. Mice

*Arhgef2^-/-^* C57BL/6 mice were generated as previously described [24] and were obtained from Hans-Christian Reinecker (Massachusetts General Hospital, Harvard Medical School). WT C57BL/6 mice were obtained from the National Laboratory Animal Center (NLAC), NARLabs (Tainan, Taiwan). All animals were bred and housed at National Taiwan University Animal Resource Center under specific pathogen-free (SPF) conditions according to institutional guidelines. All experiments were carried out on female mice at 8- to 12-week old with protocols approved by the Institutional Animal Care and Use Committee (IACUC) at National Taiwan University (approval code: NTU-103-EL-68, 1 November 2014).

### 4.2. Bone Marrow-Derived Macrophages, COS-7 Cells, and Plasmid

Bone marrow-derived macrophages (BMDM) were generated by flushing bone marrow cells from femurs and tibia of WT and *Arhgef2^-/-^* mice followed by depleting erythrocytes using Ammonium-Chloride-Potassium (ACK) lysis buffer (Gibco), passing cells through a 70 μM cell strainer. Resuspending cells in DMEM (Gibco) supplemented with 10% FBS (Gibco), 1% penicillin/streptomycin (Gibco), and 20 ng/mL M-CSF (eBioscience). COS-7 cells were purchased from Bioresource Collection and Research Center (BCRC) and cultured by DMEM supplemented with 10% FBS (Gibco) and 1% penicillin/streptomycin (Gibco). Cells were maintained at 37 °C, 5% CO_2_ for six days before experiments. Plasmid encoding GEF-H1 (pCMV6-Entry-hGEF-H1) was purchased from Origene.

### 4.3. Treatments and Measurement of Il-1β

WT or GEF-H1-deficient BMDMs were seeded in 6-well plates at 2 × 10^6^/well with serum-free DMEM overnight at 37 °C, 5% CO_2_. BMDMs were then primed with or without 100 ng/mL LPS (Invivogen) in serum-free DMEM at 37 °C, 5% CO_2_ for 6 h followed by incubation with the inflammasome activators for 6 h: 1 μg/mL poly(dA:dT) (Invivogen) complexed with lipofectamine 2000 (Invitrogen), 1 μg/mL flagellin (Invivogen) complexed with lipofectamine 2000 (Invitrogen), 5 μM nigericin (Invivogen) complexed with lipofectamine 2000 (Invitrogen), or 0.4 mg/mL alum crystal (Invivogen) (Invitrogen). In the disruption of cytoskeleton experiments, BMDMs were first pretreated with 10 μM nocodazole (Sigma), 10 μM colchicine (Sigma), or 5 μM cytochalasin D (Sigma) for 1 h followed by the aforementioned stimulus. BMDM cell supernatants were collected and centrifuged at 3000 rpm for 5 min to remove debris. The concentration of Il-1β in supernatants was measured using commercial enzyme-linked immunosorbent assay (ELISA) kits (R&D system) based on the manufacturer’s instructions.

### 4.4. Immunoblotting

BMDMs were lysed as previously described [24] and protein concentrations were measured by BCA protein assay kit (Pierce). Cleared lysates were separated by 12% SDS-PAGE followed, transferred onto PVDF membranes (Millipore) and then blocked for 1 h at room temperature with 5% nonfat dried milk in PBST (phosphate-buffered saline, 0.1% Tween-20). Membranes were then probed with antibodies against GEF-H1 (abcam, Cat. #ab155785, 1:1000), NLRP3 (Adipogen, Cat. # AG-20B-0014-C100, 1:2000), ASC (santa cruz, Cat. #sc-22514-R, 1:1000), caspase-1 (santa cruz, Cat. #sc-514, 1:1000), acetylated α-tubulin (cell signaling, Cat. #5335, 1:1000), total α-tubulin (abcam, Cat. #ab4074, 1:1000) and β-actin (cell signaling, Cat. #3700, 1:10,000) for 16 h at 4 °C. Peroxidase-conjugated goat anti-rabbit (Jackson ImmunoResearch Laboratories, Cat. #111-035-003, 1:10,000), goat anti-mouse (Jackson ImmunoResearch Laboratories, Cat. #115-035-003, 1:10,000), donkey anti-goat (Jackson ImmunoResearch Laboratories, Cat. #705-035-003, 1:10,000) antibodies were used as secondary antibodies according to the sources of first antibodies for 1 h at room temperature.

For detection of cleaved caspase-1 in BMDM supernatants. Supernatants were collected and transferred into Vivaspin 500 Protein Concentrator columns (GE Healthcare) and centrifuged at 14,000 rpm at 4 °C until the remaining volume in the column was less than 100 μL. The concentrated supernatants were transferred into microcentrifuge tubes and diluted the samples with 4× Laemmli Sample Buffer (BioRAD). The volume of each sample was adjusted equally by adding 1× Laemmli Sample Buffer (BioRAD). Samples were separated by 14% SDS-PAGE followed by immunoblotting using an antibody against caspase-1 (Adipogen, 1:1000) for 16 h at 4 °C. Peroxidase-conjugated goat anti-mouse (Jackson ImmunoResearch Laboratories, 1:10,000) was used as a secondary antibody for 1 h at room temperature. Uncropped images are shown in the Supplementary Figures S2–S6.

### 4.5. Detection of Lactate Dehydrogenase (LDH) Release

Cell death was assessed by measuring the release of LDH in BMDMs. Released LDH in BMDM cell culture supernatants was analyzed by CytoTox96 cytotoxicity assay (Promega). The percentage of LDH release was calculated as 100 × [(experimental LDH release; medium background) / (maximum LDH release; medium background)].

### 4.6. Quantitative RT-PCR (qRT-PCR)

Total RNA from WT and GEF-H1-deficient BMDMs was extracted by TRIzol (Invitrogen). 1 μg of total RNA was reverse transcribed to cDNA using iScript cDNA synthesis kit (Bio-Rad). The expression of *Il1b* was performed using iQ SYBR green supermix (Bio-Rad) and analyzed on CFX96 real-time PCR detection system (Bio-Rad). The mRNA level of *Nlpr3, Pycard, Casp1* was normalized to that of *Gapdh*. Primers used for qRT-PCR analysis were listed as follows: *Nlrp3*, forward, 5′-GCTCCAACCATTCTCTGACC-3′ and reverse, 5′-AAGTAAGGCCGGAATTCACC-3′. *Pycard*, forward, 5’-AGGAGTGGAGGGGAAAGC-3’ and reverse, 5’-AGAAGACGCAGGAAGATGG-3’, *Casp1*, forward, 5’-AGGAATTCTGGAGCTTCAATCAG-3’ and reverse, 5’-TGGAAATGTGCCATCTTCTTT-3’, *Gapdh,* forward, 5′-AACTTTGGCATTGTGGAAGG-3′ and reverse, 5′-GGATGCAGGGATGATGTTCT-3′.

### 4.7. Confocal Microscopy

WT or *Arhgef2^-/-^* BMDMs were seeded into 12 mm circular cover glasses (Karl Hecht Assistant) primed with LPS, stimulated with indicated inflammasome inducers followed by staining with Mitotracker Deep Red FM (Invitrogen). Images were acquired with SP8 LIGHTNING confocal microscope (LEICA).

### 4.8. Bacterial Infection and Gentamycin Protection Assay

*Salmonella typhimurium* SL1344 was grown in LB at 37 °C overnight. WT or *Arhgef2^-/-^* BMDMs were seeded in 6-well plates and spin-infected with *Salmonella typhimurium* at the multiplicity of infection (MOI) of 25 for 800 g, 15 min at room temperature followed by incubation at 37 °C for 45 min. Cells were washed twice before the addition of 100 μg/mL gentamicin (Sigma) in DMEM with 10% FBS for 1 h to eliminate extracellular bacteria. Cells were then given fresh DMEM with 10% FBS and 10 μg/mL gentamicin (Sigma) for the rest of the experiment. To determine the intracellular replication of bacteria, cells were lysed by 1% Triton X-100 (Sigma) in PBS. Serially diluted cell lysates were spread on LB agar plates and incubated at 37 °C for 16 h for colony enumeration.

### 4.9. Statistical Analysis

Two-tailed unpaired student’s t-test or one-way analysis of variance (ANOVA) were carried to determine the statistical significance of the experimental results. A *p*-value < 0.05 was considered statistically significant.

## Supplementary Materials

Supplementary materials can be found at https://www.mdpi.com/1422-0067/21/4/1302/s1.

## Abbreviations

PRR	Pattern recognition receptor
NLRP3	NLR family pyrin domain-containing protein 3
NLRC4	NLR family CARD domain-containing protein 4
NLR	NOD-like receptor
CARD	Caspase activation and recruitment domain
NOD	Nucleotide-binding oligomerization domain
NOD1	Nucleotide-binding oligomerization domain-containing protein 1
NOD2	Nucleotide-binding oligomerization domain-containing protein 2
AIM2	Absent in melanoma-2
NEK7	NIMA-related kinase 7
NIMA	Never in mitosis gene a
MARK4	Microtubule-affinity regulating kinase 4
GEF-H1	Guanine nucleotide exchange factor-H1
WT	Wild-type
BMDM	Bone marrow-derived macrophage
LPS	Lipopolysaccharide
SPI-1	*Salmonella* pathogenicity island 1
T3SS	Type 3 secretion system
RLR	RIG-I-like receptor
