# Supplementary material for: Microtubule-Mediated NLRP3 Inflammasome Activation Is Independent of Microtubule-Associated Innate Immune Factor GEF-H1 in Murine Macrophages
Source: Int J Mol Sci. 2020 Feb 14;21(4):1302. doi: 10.3390/ijms21041302 (PMC7072875; doi:10.3390/ijms21041302)
Supplement: Supplementary file 1 [file ijms-21-01302-s001.pdf]

Supplementary materials

Microtubule-mediated NLRP3 inflammasome activation is independent of microtubule-associated innate immune factor GEF-H1 in murine macrophages

Hsuan-Ju Lai [1, †], Yi-Hsuan Hsu [1, †], Guan-Ying Lee [1, †] and Hao-Sen Chiang [1, 2, *]

[1] Department of Life Science, National Taiwan University, Taipei 10617, Taiwan

[2] Genome and Systems Biology Degree Program, National Taiwan University, Taipei 10617, Taiwan

[†] These authors contributed equally to this work

[*]Corresponding author:

Hao-Sen Chiang, Ph.D.

R818 Life Science Building

1 Sec 4 Roosevelt Road

Taipei 10617, Taiwan

Phone: +886-2-3366-2454

Fax: +886-2-2363-6837

E-mail: hschiang@ntu.edu.tw

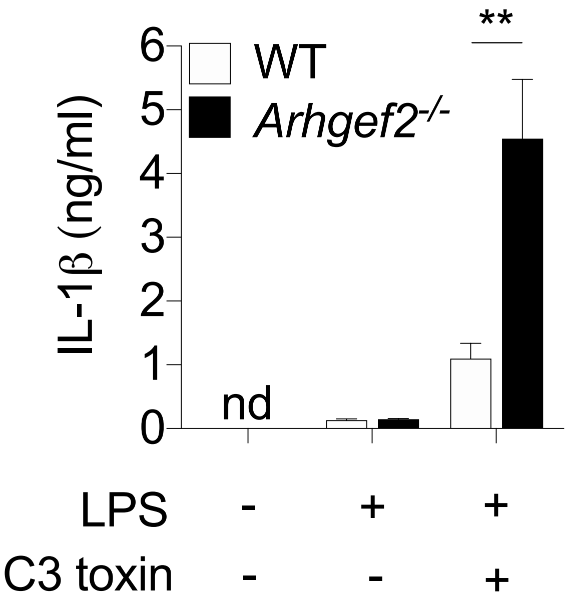

Supplementary Figure 1. GEF-H1 regulates the pyrin inflammasome activation. ELISA analysis of culture supernatants of LPS-primed wild-type (WT) or GEF-H1-deficient (*Arhgef2*$^{-/-}$) BMDMs treated with or without C3 toxin. BMDMs were first primed with 100 ng/ml LPS for 6 h followed by incubation with 0.5 µg/ml C3 toxin (Cytosekleton, Cat. #CT03) for additional 6 h. n=3 independent experiments per group. Data represent mean±SD. Statistical analyses were performed using unpaired two-tailed t-test. **, p<0.01.

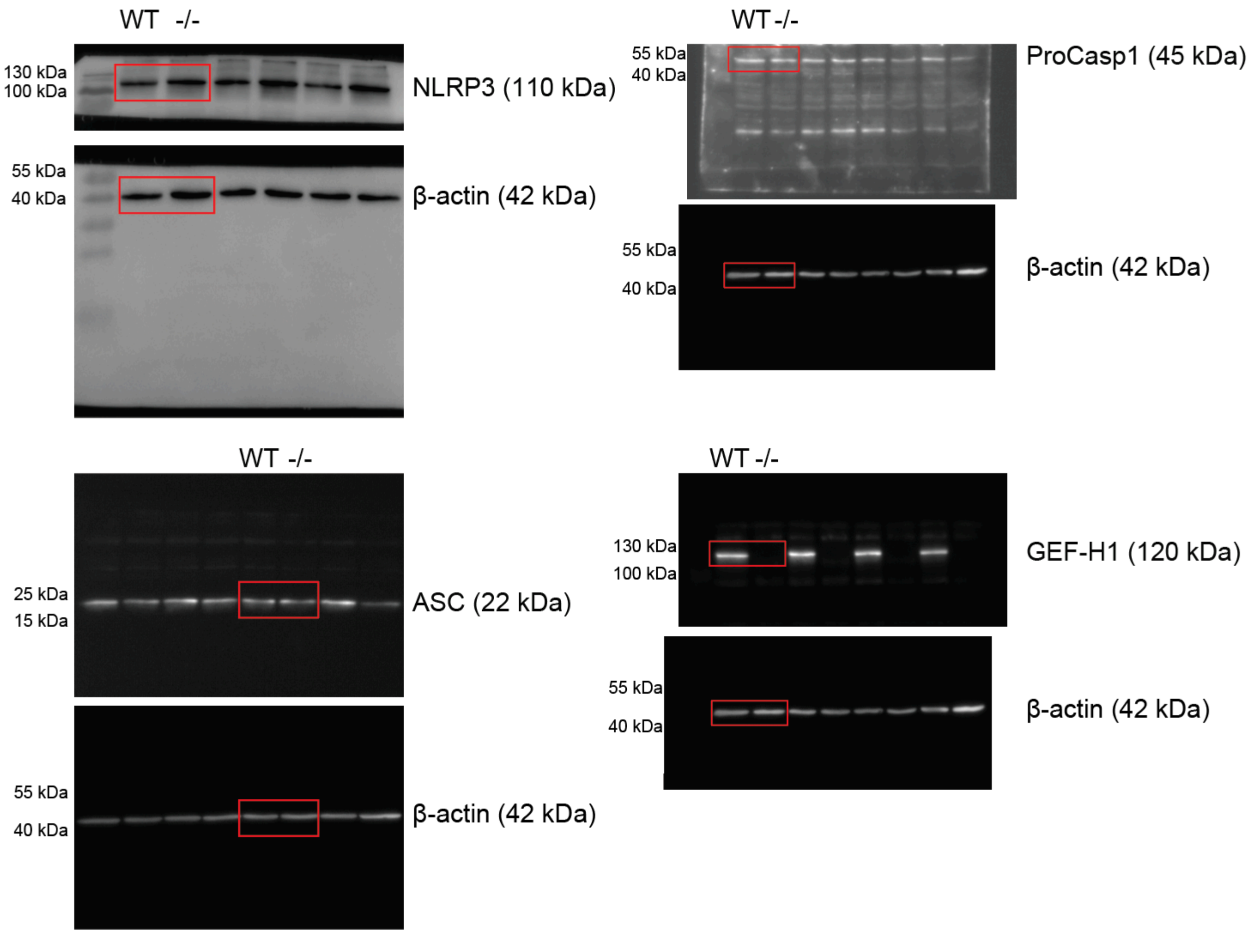

Supplementary Figure 2. Uncropped pictures of the western blots shown in the manuscript in Figure 2B.

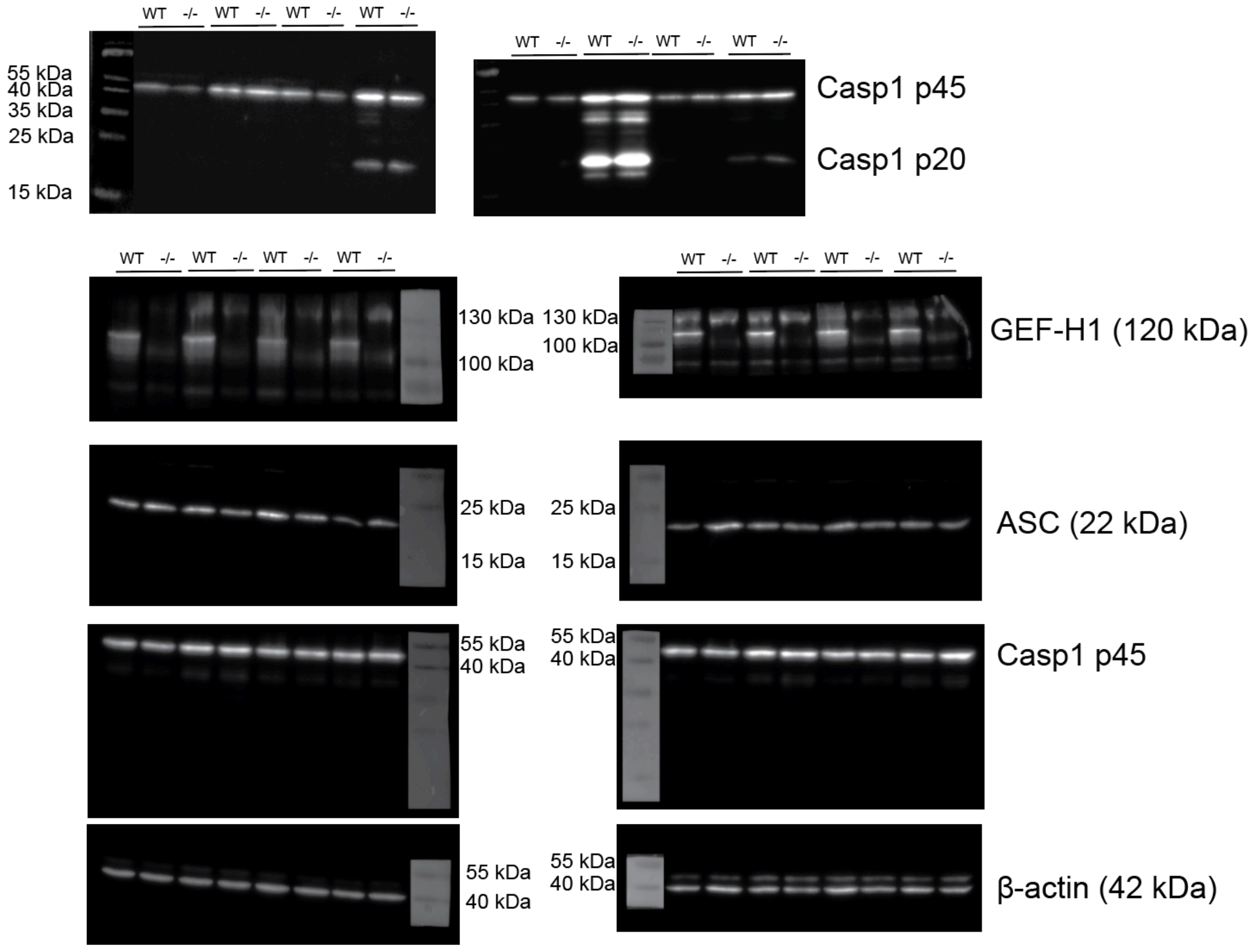

Supplementary Figure 3. Uncropped pictures of the western blots shown in the manuscript in Figure 2D.

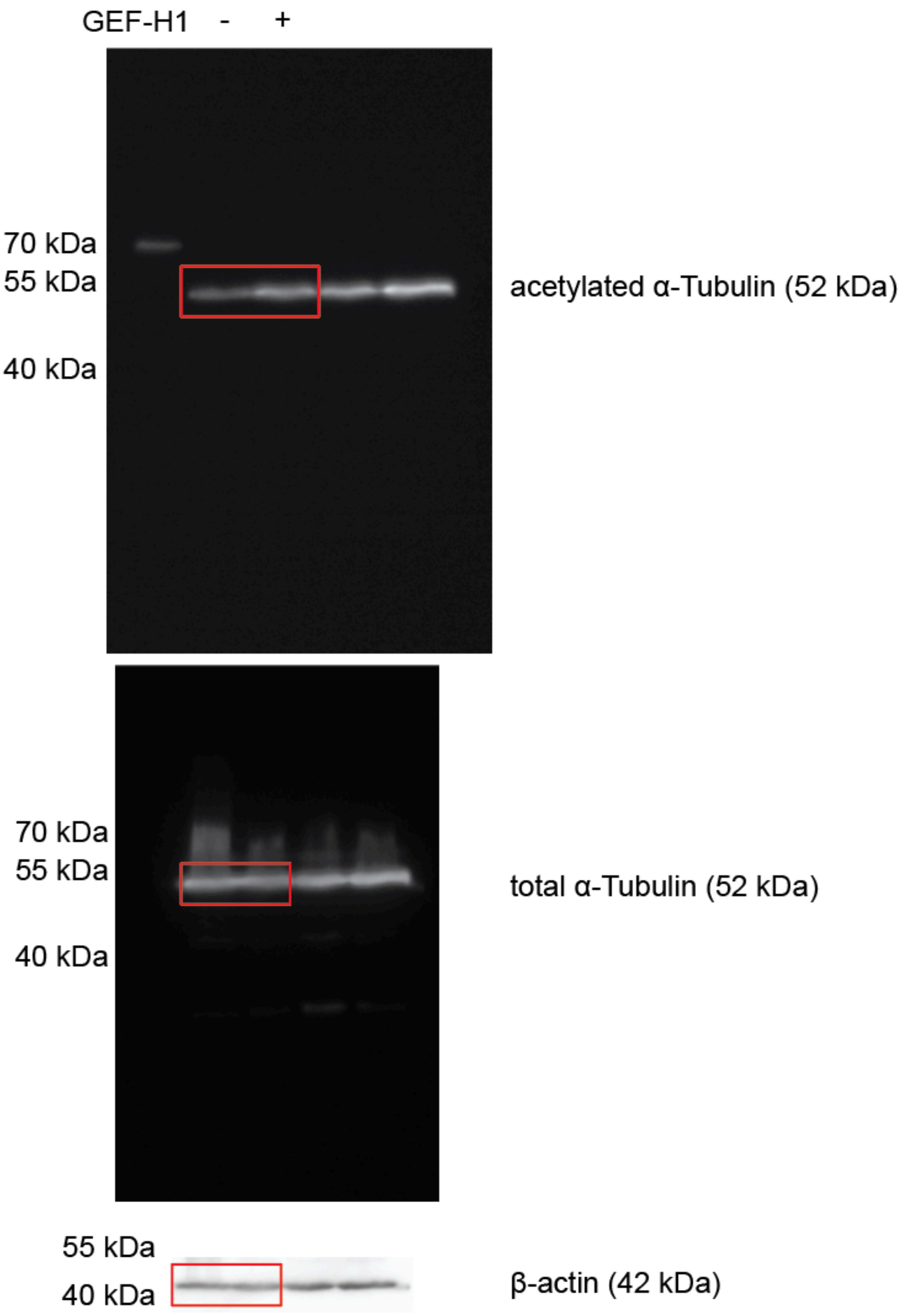

Supplementary Figure 4. Uncropped pictures of the western blots shown in the manuscript in Figure 3A.

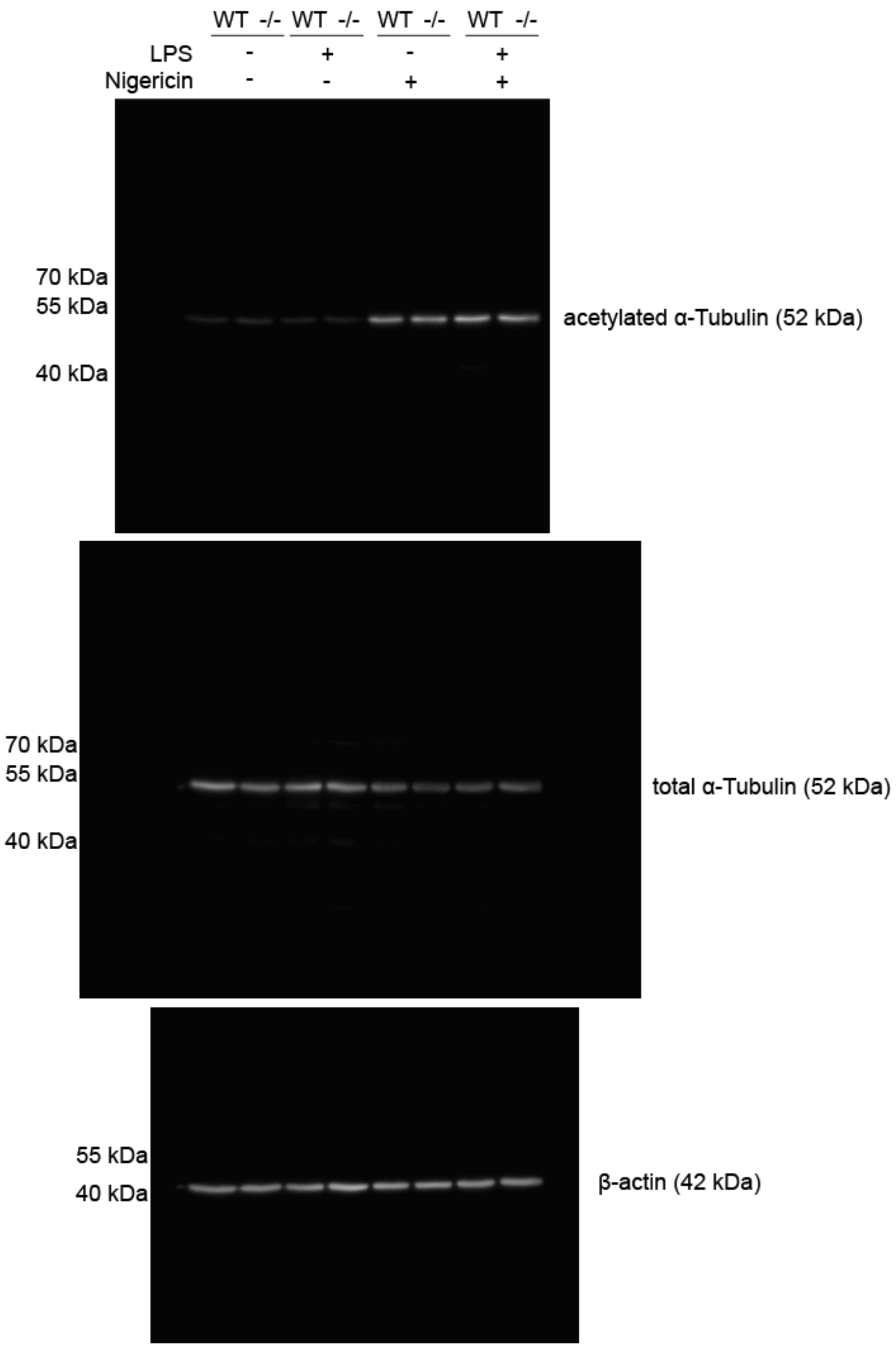

Supplementary Figure 5. Uncropped pictures of the western blots shown in the manuscript in Figure 3B.

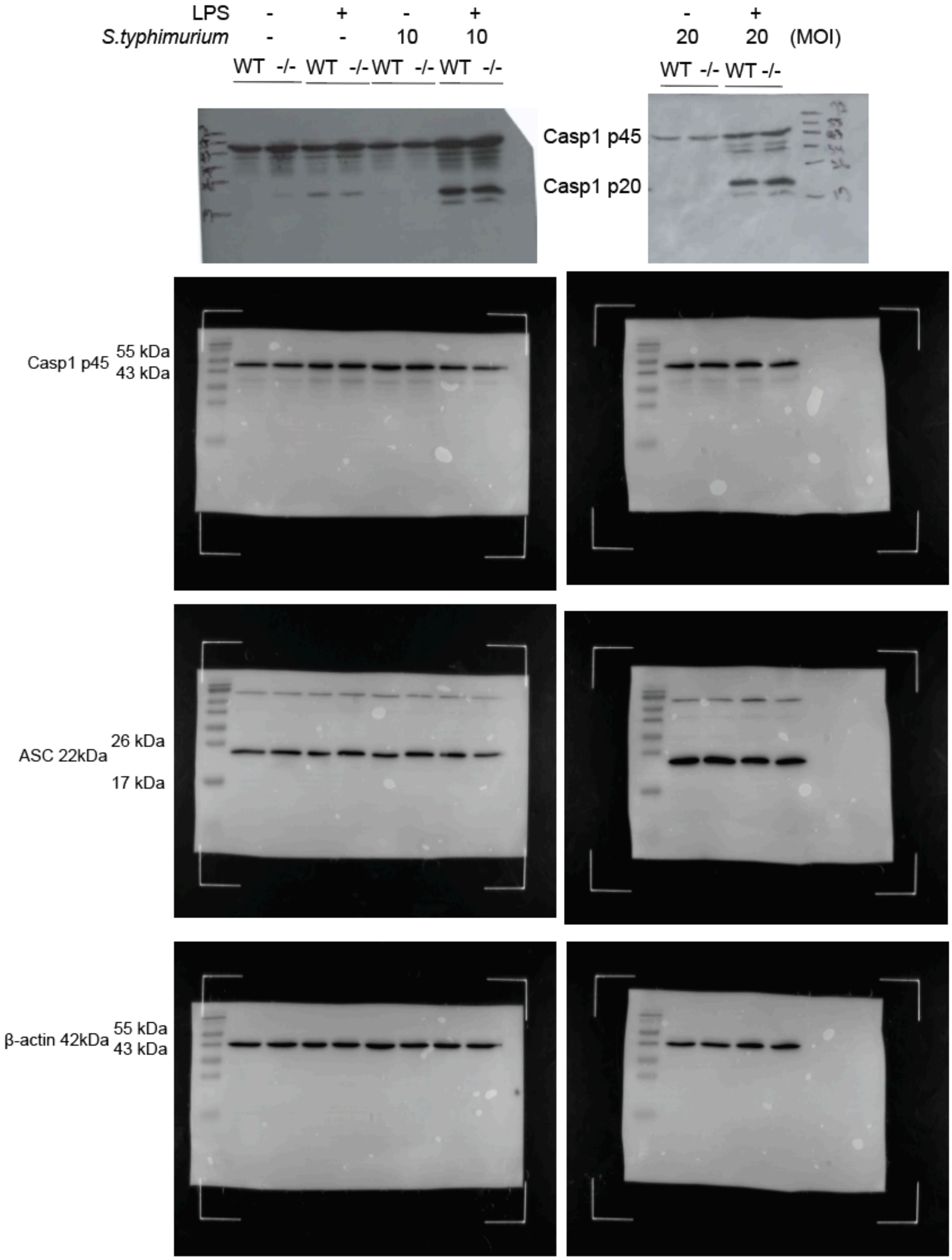

Supplementary Figure 6. Uncropped pictures of the western blots shown in the manuscript in Figure 4B.
